# Anti-staphylococcal, anti-HIV and cytotoxicity studies of four South African medicinal plants and isolation of bioactive compounds from *Cassine transvaalensis* (Burtt. Davy) codd

**DOI:** 10.1186/1472-6882-14-512

**Published:** 2014-12-18

**Authors:** Ningy S Mthethwa, Bola AO Oyedeji, Larry C Obi, Olayinka A Aiyegoro

**Affiliations:** Department of Medical Microbiology, Walter Sisulu University, Private Bag X1, Mthatha, 5099 South Africa; Department of Chemistry and Chemical Technology, Walter Sisulu University, Private Bag X1, Mthatha, 5099 South Africa; GI Microbiology and Biotechnology Unit, Agricultural research Council- Animal Production Institute, Private Bag X02, Irene, 0062 South Africa; Division of Research and Academic Affairs, University of Fort Hare, Private Bag X1314, Alice 5700, Eastern Cape Province, South Africa

**Keywords:** Staphylococci, Medicinal plant, Cytotoxic, Disk diffusion, Bioactive

## Abstract

**Background:**

Medicinal plants represent an important opportunity to rural communities in Africa, as a source of affordable medicine and as a source of income. Increased patient awareness about safe usage is important as well as more training with regards to traditional medicine. The aim of this study was to evaluate the ethnomedicinal prowess of some indigenous South African plants commonly used in Eastern Cape Province of South Africa for the treatment of skin and respiratory tract infections, HIV and their toxicity potential.

**Methods:**

*Cassine transvaalensis*, *Vangueria infausta*, *Croton gratissimus* and *Vitex ferruginea* were tested for antibacterial activities against *Staphylococcus aureus* and *Staphylococcus epidermidis* using Kirby-Bauer disk diffusion and minimum inhibition concentration (MIC). Cytotoxic and anti-HIV-1 activities of plants were tested using MTT Assay (3- (Dimethylthiozole-2-yl-2,5-diphenyltetrazolium bromide)) and anti- HIV-1iib assay. In search of bioactive lead compounds, *Cassine transvaalensis* which was found to be the most active plant extract against the two *Staphylocoous* bacteria was subjected to various chromatographic. Thin layer chromatography, Column chromatography and Nuclear Magnetic Resonance (NMR), (^1^H-^1^H, ^13^C-^13^C, in DMSO_d6, Bruker 600 MHz) were used to isolate and characterize 3-Oxo-28-hydroxylbetuli-20(29)-ene and 3,28-dihydroxylbetuli-20(29)-ene bioactive compounds from *C. transvaalensis.*

**Results:**

The four plants studied exhibited bioactive properties against the test isolates. The zones of inhibition ranged between 16 mm to 31 mm for multi-drug resistant staphylococci species. MIC values varied between 0.6 and 0.02 μg/ml. *C. gratissimus* and *C. transvaalensis* exhibited the abilities to inhibit HIV-1_iib_. Two bioactive compounds were isolated from *C. transvaalensis.*

**Conclusion:**

Data from this study reveals the use of these plant by traditional healers in the Eastern Cape. Furthermore, *C. transvaalensis* and *C. gratissimus* were found to be more active as against HIV-1iib. While *C. transvaalensis* was most active against the two *Staphylococcus* bacteria.

## Background

Plants have been used as medicine to manage both animals and humans’ health throughout history, and studies have shown that wild animals eat certain plants to treat themselves from certain ailments [1–3]. In all the continents of the world, medicinal plants are consumed by humans for management of health related issues, although the practices and mode of uses may differ from country to country and from tribe to tribe [4].

Most Africans still depend on and prefer traditional medicine for the management of their health, just because of the fact that traditional medicine is affordably cheap, available and accessible, also because of the folkloric beliefs that traditional herbal medicine has no side effects [5]. Change in life style and behaviour have contributed to the loss of knowledge concerning medicinal plants. For many years people have used ingredients derived from plants to treat various illnesses.

*C. gratissimus* Burchell is a shrub that may grow up to 10 metres in height. It is a slender tree with fine drooping foliage and a crown which spread in a V-shape with drooping terminal branches [6]. The upper surface of the leaves is dark-green and shiny without hairs, while the under surface is covered with scales resulting in silvery colour. It is found over a wide range of altitudes in a variety of wood land type, often associated with rocky outcrops [6]. The bark powder is used to treat bleeding gums [7]. Milk infusions of the bark are used as purgatives for stomach disorders [6, 7]. In Botswana, the leaves are used for treating coughs [6, 7], while Zimbabweans, use the roots infusions for healing abdominal pains [7].

*C. transvaalensis*is Burth- Davy is a densely branched, untidy tree with a roundish canopy [8]. The bark and branches of the younger trees are light in color with fairly smooth while the older branches and stem are darker with rougher patches. The leaves on the older trees are light green in color and are arranged in groups on very short twigs coming off the branches at right angles [8, 9]. The bark of the tree is used for tanning and to make tea. It is said to be an excellent treatment for stomach problems. It is also used as an enema for stomach ache and fever and to treat diarrhoea. The leaves are chewed and juice swallowed for sore throat [9].

*V. infausta* Burch is a small tree that varies in height from 3–7 m, depending on the habitat [9]. It can be single or multi-stemmed, but usually the latter. The bark is greyish to yellowish brown, smooth and peeling in irregular small strips [6]. The branchlets are covered with short, woolly hairs, especially when young [6, 9]. An infusion of leaves and roots is used to treat ringworms, toothache, malaria and chest ailments like pneumonia [10].

*V. ferruginea* Schumach & Thonn is a shrub which grows in bush veld thicket and sand forest usually on deep sand [9]. Leaves are aromatic, slender and elongated, may be narrowly ovate or elliptic, with the upper surface smooth or slightly hairy on veins and rusty velvety below [9]. The decoction of *V. ferrugenea* is known to be used traditionally for treating coughs, chest pains and stomach disorders [9].

The uncensored uses of anti-infective agents have resulted in emergence of drug resistant bacteria, fungi and viruses [11], with staphylococcus species taking the leads of most reported emerging resistant bacterial specie. Among the staphylococci, *Staphylococcus aureus* and *Staphylococcus epidermidis* have great pathogenic potential, with myriads of literatures citing the opportunistic virtues of these two strains in causing difficult to treat systemic infections [11–13]. Moreover *S. epidermidis* is well known for its potential to be opportunistic and to greatly initiate infections in immune-compromised patients [13].

*S. aureus* and *S. epidermidis* infections are treated with antibiotics; penicillins being the drug of choice. The percentage of staphylococcal strains exhibiting wide-spectrum resistance to antibiotics have become increasingly rampant, posing a threat for effective antimicrobial therapy [14, 15].

In a bid to find alternatives treatment of resistant Staphylococccus infections; this study was carried out to evaluate the antibacterial and anti-HIV-1_iib_ activities of four medicinal plants commonly used in Eastern Cape Province, South Africa for the treatment of skin and respiratory tract infections. Furthermore, the most active of all was subjected to bioactive isolation in a search for bioactive compounds that could be used in drug formulation. This study also intends to shed more light on the hidden biological potentials of the four candidate plants studied in this research as templates for future drug development.

## Methods

### Plant material

1. Scientific name: *Croton gratissimus* Burch.

Family: Euphorbiaceae

Xhosa name: Mahlabekufeni

Common name: Lavender croton

2. Scientific name: *Cassine transvaalensis* (Burtt. Davy) Codd

Family: Celastraceae

Xhosa name: iNgwavuma

Common name: bushveld saffron (Eng.)

3. Scientific name: *Vangueria infausta* Subsp. *Infausta* Burch

Family: Rubiaceae

Xhosa name: uMviyo

Common name: wildmedlar (Eng)

4. Scientific name: *Vitex ferruginea* subsp. *Amboniensis* Schumuch &Thonn

Family: Lamiaceae

Xhosa name: Umphenduli

Common name: Plum finger leaf

*Vangueria infausta* (roots)*, Cassine transvaalensis* (bark)*, Croton grattisimus* (leaves) and *Vitex ferruginea* (leaves) were collected near flagstaff in the Eastern Cape, South Africa and identified by Immelman KL in Botany Department of Walter Sisulu University. Voucher specimens were deposited in the herbarium of Botany Department, Walter Sisulu University, Eastern Cape, South Africa with voucher specimen numbers MNS 001, MNS 002, MNS 004, and MNS 003 for *V. infausta*, *C. transvaalensis*, *C. grattisimus* and *V. ferruginea* respectively. Plant material was air dried, ground and extracted with distilled methanol. Plants material (1.5 kg) was soaked and placed on the shaker for two days, filtered with Whatmanns No1 filter paper then concentrated using rotary evaporator [16].

### Bacterial inoculum and magi cells culture preparation

One hundred and eighty three multidrug resistant hospital isolates of staphylococcus strains: *S. aureus* (100), *S. epidermidis* (83) were used in this study. American Type Culture Collection (ATCC) strains of *S. aureus* (25923) and *S. epidermidis* (12228) were used as controls. *S. aureus* and *S. epidermidis* strains were grown on Mueller Hinton broth (Oxoid) medium at 37°C. The culture was then adjusted to 0.5 McFarland standard.

MAGI CCR5 cell lines purchased from ATCC, Manassas, VA 20108, USA were used for cytotoxic screening of medicinal plant extracts. Trypsin was added to magi cells and incubated for 5 minutes to detach them from each other. The cells were then diluted with Dulbecco’s modified eagle medium (DMEM) with L-Glutamin and Phenol red media. Cell lines were cultured in advanced Modified Eagle’s Medium with 10% 5mML-glutamine (GibcoBRL) and grown at 37°C in a 5% CO_2_ humidified incubator (Thermo Fisher Scientific, Wakenyaku Co. Ltd, Japan). Cells were sub culture devery 48 hours until confluent growth was observed.

### Antibacterial screening

#### Kirby-bauer disk diffusion

One gram of each plant extract was reconstituted in 10% Dimethyl sulphoxide (DMSO) to get a concentration of 0.1 g/ml. This was further diluted to 0.05 g/ml. *C. transvaalensis* (bark), *V. infausta* (roots), *C. grattisimus* (leaves) and *V. ferruginea* (leaves) extracts were tested against different strains of *S. aureus* and *S. epidermidis*. Fifteen microliters (15 μl) of plant extract was suspended on a 6 mm paper disks and placed on Mueller-Hinton agar plate inoculated with *S. aureus* or *S. epidermidis*. The plates were then incubated at 37°C for 18 hours. The antibacterial activities were evaluated by measuring inhibition zone diameters [17]. These assay was done in triplicates.

### Minimum Inhibitory Concentration (MICs) assay

A micro-dilution technique described by [18] which uses 96-well microtitre plate was used for MICs. The microbial cultures were diluted in fresh Mueller-Hinton broth to a 0.5 McFarland standard. Fifty microliters of Mueller Hinton broth was added to all wells, followed by 50 μl broth culture and 50 μl of plant extracts dissolved in DMSO. Plant extracts were diluted from the first row of the micro-titre plate. Cloxacillin was used as positive control and DMSO as negative control. Micro plates were covered with lids and incubated at 37°C overnight. Fifty microliters of 0.2% solution of P-iodonitrotetrazolium violet (Sigma) (0.2 mg/ml) reagent was used to indicate the presence of uninhibited bacterial growth (purple colour) or inhibition (colourless) of bacterial growth in each well. The lowest concentration of crude plant extract that inhibited bacterial growth was taken as the MIC [18]. These assay was done in triplicates.

### Cytotoxic and anti- HIV-1 screening of medicinal plants

#### MTT assay 3- (Dimethylthiozole-2-yl-2, 5-diphenyltetrazolium bromide)

MTT assay was performed to determine cytotoxicity of plant extracts as first described by Mosmann [19] with modifications suggested by Akhir [20]. MAGI CCR5 cells were seeded into two 96 well plates with 10^4^ cells/well in 100 μl of DMEM supplemented with 10% foetus bovine serum (FBS). Eleven microliters (5 mg/ml) of plant extract was added. After 48 h cells were observed and supernatant from each well discarded. The MTT reagent (10 μl) was added into each well. Plates were incubated at 37°C for 4 h, after which 100 μl MTT-stop solution containing sodium dodecyl sulphate was added into each well and plates optical density was observed at a wave length of 570 nm then the 50% cytotoxic concentration (CC_50_) was determined. A toxic substance, berberine was used as a positive control [21].

### Anti-HIV-1_IIIB_ assay

Anti-HIV-1 assay was conducted according to Kanamoto [22]. The inhibitory effects of the plant extracts on HIV-1_iib_ replication were estimated by the levels of inhibition of virus induced cytopathic effects (CPE) in MT-4 cells. MAGI CCR5 cells in Dulbecco’s modified Eagle medium containing 10%FBS and plant extracts were cultured in 96 well culture plate for 24 hrs. One hundred microliters of the HIV-1virus was added into wells. After two days the cells were fixed with 100 μl of phosphate buffered saline (PBS) containing 1% formaldehyde and 0.2% gluteraldehyde for 5 min at room temperature. The cells were washed three times with PBS and incubated for 1 h at 37°C with 100 μl of staining solution (4 mM potassium ferrocyanide, 4 mM potassium ferricyanide, 2 mM MgCl_2_, and 400 μg of 5-bromo-4-chloro-3-indolyl-β-D-galactoside per ml). The reaction was stopped by washing twice the stain off with PBS and finally100 μl of PBS was added into the wells. Cells infected with HIV, the integrated HIV long terminal repeat (LTR) β-gal reporter gene was expressed and the cells turned blue on staining. Blue cells were counted under light microscope. The 50% effective concentration (EC_50_) and selectivity index (SI = CC_50_/EC_50_) were determined. The anti-HIV-1 activity of the plant extract was represented as the percent inhibition of the blue cell expression and was calculated as follows:


Infection with virus (1:5 v/v of virus/media) to obtain a 200X TCID (Tissue culture infective dose) was considered adequate for this study. All tests and analyses were run in triplicate.

### Isolation of active compounds

#### Sequential solvent extraction

Hexane, dichloromethane (DCM), ethyl acetate, methanol were used in the sequential extraction respectively. The extracts were filtered using Whatmanns no1 filter paper, then the solvents were removed by vacuum distillation in a Buchi rotary evaporator. The extracts were concentrated such that almost all the extracting solvent was removed then transferred to pre-weighed beakers and allowed to dry completely in the fume cupboard. The beakers were weighed again in order to determine the actual weight of the extracted material.

### Thin layer chromatography TLC

The dried plant extracts were re-dissolved in methanol. Each plate was spotted with two plant extracts using the capillary tube. The separation was carried out at room temperature on a 10 × 10 cm silica gel plates (ALUGRAM® SIL G fűr die DC). The chromatograms were run in small glass tanks with the running solvent of hexane: ethyl acetate (80:20). The plates were dried at room temperature and developed in crystal iodine.

### Bio-autographic assay

Methanol plant extracts were spotted on the TLC plate (ALUGRAM® SIL G fűr die DC) using capillary tube and separated in 80:20 (hexane: ethylacetate) as it was found to be the best separating solvent for *Cassine transvaalensis.* The plate was allowed to dry at room temperature. The test culture was sprayed on the TLC plate and incubated at 37°C overnight. The following day the plates were sprayed with iodonitrotetrazolium (INT) (0.2 mg/ml) [23]. The locations of the active compounds were numerically presented by calculating the retention factor (R_f_).

### Column chromatography for separating plant compounds

A 400 ml glass column was packed with 60 g of fine silica gel (1.10757.1000). Two grams of plant extract to be run in the column was mixed with silica gel and ground to fine powder, this was then poured on top of the sand layer. 200 ml of 100% hexane was first used to run the column, followed by 200 ml of hexane: ethyl acetate (95:5), the polarity was gradually increased with ethyl acetate until the ratios reached 50:50. Twenty millilitres of fractions were collected in each beaker, and spotted on the TLC plate. Hexane: ethyl acetate (80:20) was used as a mobile phase to run TLC. Developing spots were observed under a 250 nm wavelength UV light and in crystal iodine vapour. The fractions with the same bands appearing on the TLC plate were combined. The R_f_ values were calculated and compared to those found in the bioassay plates for the active compounds.

Spectroscopic data analysis, NMR (1H-1H, 13C-13C, in DMSO_d6, Bruker 600 MHz) technique was used to confirm the structure of compounds. Chemical shifts were expressed in δ (ppm).

## Results

### Antibacterial susceptibility against medicinal plants

The four plant extracts used in this study demonstrates good to moderate bioactivities as confirmed by different assays in the study. The plant extracts (0.1 g/ml) exhibited antibacterial activities against multidrug resistant *S. aureus* and *S. epidermidis* strains isolated from patients (Table 1). The zones of inhibition ranged between 16 mm to 31 mm for multi-drug resistant staphylococci species. *C. transvaalens is* exhibited the average of 31 mm zone of inhibition against *S. epidermidis* and 23 mm against *S. aureus. V. ferruginea* seemed to have lower average zones of inhibition against *S. aureus* (16 mm) and *S. epidermidis* (19 mm). It was observed that the ATCC control strains of *S. epidermidis* (12228) and *S. aureus* (25923) exhibited zones of inhibition ranging from 17 mm to 25 mm.

**Table 1:** **Average zones of inhibition exhibited by the plant extracts against** ***Staphylococci*** **isolates**

Plant extract (0.1 g/ml)	Average zones of inhibition (mm)		ATCC (25923) ***S. aureus***	ATCC (12228) ***S. epidermidis***
	***S. aureus***	***S. epidermidis***		
*C. transvaalensis*	23	31	23	25
*V. infausta*	23	24	20	23
*C. gratissimus*	22	27	20	25
*V. ferruginea*	16	19	17	20

The minimum inhibition concentration (MIC) of the crude extracts was also determined. MIC values varied between 0.6 and 0.02 μg/ml (Table 2). *C. transvaalensis* was observed to be the most bioactive plant extracts which was able to inhibit 6% multi drug resistant strains of *S. aureus* and 2% *S. epidermidis* from patients at a minimum concentration of 0.02 μg/ml. *C. gratissimus* inhibited 17% and 5% *S. aureus* and *S. epidermidis* multidrug resistant strains at a concentration of 0.2 (μg/ml). MIC values of *V. infausta* were higher than the values represented in Table 2.

**Table 2:** **Minimum inhibition concentration (MIC) of plant extracts against** ***S. aureus*** **and** ***S. epidermidis***

MIC (μg/ml)	***C. transvaalnsis***(%)		***V. infausta***(%)		***C. gratissimus***(%)		V. ferruginea (%)	
	***S. aur***	***S.epi***	***S. aur***	***S.epi***	***S. aur***	***S. epi***	***S. aur***	***S.epi***
0.6	4	17	6	6	4	33	ND	ND
0.2	22	31	48	28	5	17	ND	ND
0.06	12	6	14	n.a	n.a	n.a	ND	ND
0.02	6	2	3	n.a	n.a	n.a	ND	ND

Table legend: *S. aur = S. aureus*; *S. epi = S. epidermidis; ND = Not Determined; n.a = Not applicable.*

### Anti-HIV-1 and cytotoxic activities results

*C. gratissimus* and *C. transvaalensis* exhibited abilities to inhibit HIV-1_IIIB_ and displayed SI values >5 which are considered significant. The evaluation of cytotoxic and anti-HIV activities by MTT assay resulted in CC_50_ of 200 μg/ml,100 μg/ml, 100 μg/ml and 130 μg/ml for *C. transvaalensis*, *C. gratissimus, V. infausta* and *V. ferrugenea* respectively which were higher than that of the positive control (berberine).The EC_50_ of *C. gratissimus* and *C. transvaalensis* was estimated to be 9.6 μg/ml and 3.5 μg/ml with selectivity indexes (SI) of 10.4 and 57.1 respectively indicating anti-HIV-1_iib_ (Tables 3 and 4).

**Table 3:** **Anti-HIV and cytotoxic activities determined by MTT and MAGI assays**

Plant extract (mg/ml)	EC _50_(μg/ml)	CC _50_(μg/ml)	SI
*C. gratissimus*	9.6	100	10.4
*C. transvaalensis*	3.5	200	57.1
*V. infausta*	n.a	100	n.a
*V. ferrugenea*	n.a	130	n.a
Berberine	n.a	27	n.a

Table legend: *n.a = Not applicable,* SI = Selectivity index.

**Table 4:** ^**1**^ **H-NMR and** ^**13**^ **C-NMR data of compound 1 and 2 compared to literature**

	^1^H (600 MHz)		^1^H ^lit^	^13^C (600 MHz)		^13^C ^lit^	
	CPD 1	CPD 2	CPD 1	CPD 1	CPD 2	CPD 1 [24]	CPD 2 [24]
1	0.88(1H,t)	0.64(1H,s) 1.21(1H,t)	0.98(m)	39.4(*t*)	38.0	39.9	38.8
	1.24(1H,t)						
2	1.98(2H,m)		1.05(1H.m)	34.4(*t*)	27.4	34.1	27.2
3	-	4.26(1H,m)	1.83(1Hm)	217.2(*s*)	74.3	218.3	78.9
			1.96(1Hm)				
4			-	47.8(*d*)	38.7	47.2	38.9
5			0.68(t)	54.5(*d*)	56.5	54.5	55.3
6			1.40(m)	19.8(*t*)	18.4	19.8	18.3
			1.6(m)				
7			1.35(m)		34.2	36.9	34.3
			1.41(m)				
8			-	43.1(*s*)	39.8	42.4	40.9
9			1.68(m)	49.6	50.1	50.3	50.4
10			-		37.9	37.1	37.2
11			1.26(m)	21.4	22.5	21.5	20.9
			1.42(m)				
12			1.41(m)	26.2	25.8	25.2	25.3
			1.52(m)				
13			0.69(m)	37.6	33.3	37.0	37.3
14			-	47.0	44.1	47.8	42.7
15			1.41(m)	63.3	26.4	69.0	27.0
			1.52(m)				
16			1.33(m)		28.5	40.3	29.2
			1.42(m)				
17			-		48.6	47.9	47.8
18			1.77(dd)		50.9	48.8	48.8
19			3.01(dt)		48.4	47.2	47.8
20			-	155.5	150.5	149.9	150.6
21			1.41(m)		29.0	30.0	29.8
			1.51(m)				
22			1.35(m)	33.5	31.5	33.9	34.0
			1.41(m)				
23	0.84(3H,s)	0.85(3H,s)	0.81(s)	26.9	27.5	26.6	28.0
24	0.86(3H,s)	0.86(3H,s)	0.83(s)	21.4	16.2	21.0	15.4
25	0.94(3H,s)	0.90(3H,s)	0.93(s)	14.1	18.2	16.1	16.1
26	0.99(3H,)	0.97(3H,s)	0.99(s)	16.2	16.3	16.3	16.0
27	0.78(3H,s)	0.76(3H,s)	1.02(s)	5.5	16.1	8.1	14.8
28	3.8(1H,*s*,OH)	3.58 (1H, s,OH)		62.5	63.0	61.5	60.2
29	4.8(1H,*d*) 4.7(1H,*s*)	4.85(1,m) 4.72-4.75 (1,m)	4.74(brs) 4.65(br,s)	106.1(t)	106.0	110.0	109.6
30	1.64 (s)	1.63(s)	1.69(s)	18.1(q)	20.9	19.1	19.1

Table legend: CPD1 = Compound 1; CPD 2 = Compound 2; ^1^H^lit^ = From literature; ^13^C^lit^ = From literature [24–26].

### Thin layer chromatography and bio-autographic assay results

The solvents that were found suitable for the isolation of bio-active plant compounds were ethyl acetate: dichloromethane: methanol (55:5:40) using thin layer chromatography. These solvents were used as the mobile phase in the successful isolation of two compounds from *C. transvaalensis*. The bio-autographic assay revealed clear zones of inhibition with the R_f_ values of 0.3 and 0.5which corresponded with R_f_ values of compounds that were targeted in thin layer chromatography. The clear zones indicate that these compounds are able to inhibit the growth of *S. aureus* and *S. epidermidis*.

### Compounds isolated from *Cassine transvaalensis*

Compound 1 and 2 were isolated from *C. transvaalensis*, compound 1 was isolated as the white crystal powder at 80: 20 Hexane: Ethyl acetate ratio and found to be soluble in Hexane and deuterated DMSO for NMR analysis. Compound 2 was isolated as a colourless powder at a ratio of 55:5:40 Ethyl acetate:dichloromethane:methanol in chloroform and CDCl_3_ was used for the NMR runs. Structural elucidation of compound 1 and 2 was achieved by comparing their NMR spectra data with those in literature (Table 4).

#### Compound 1: 3-Oxo-28-hydroxylbetuli-20(29)-ene

**Figure 1:**
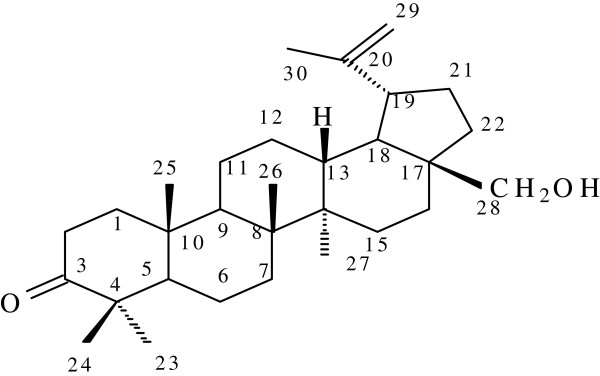
**Legend: Elucidated structure of compound 1 (3 –Oxo-28-hydroxylbetuli-20(29)-ene) [24;26]. δ**_**c**_ **(DMSO_d6)**: 14.93; 16.23; 18.18; 19.80; 21.43; 26.95; 31.82; 34.41; 35.71; 43.18; 47.08; 49.68; 54.55; 63.31; 106.18; 155.53; 217.23. **δ**_**H**_ **(DMSO_d6)**: 0.76; 0.86; 0.94; 0.99; 1.03; 1.24; 1.35; 1.35; 1.38; 1.41; 1.61; 1.64; 1.80; 1.98; 2.21; 2.23; 2.24; 2.41; 2.50; 3.90; 4.78; 4.87.

This was isolated as a white crystal powder. The ^1^H-NMR spectrum pattern suggested a triterpenoid compound. Analysis of the ^13^C-NMR spectra showed that it is a C30 compound which further strengthen the class of compound suggested. The presence of six tertiary methyl (CH_3_), and one carbonyl carbon signals in the proton and carbon 13 spectra respectively; twelve methylene (CH_2_) signals, five methane (CH) and seven quaternary carbons (C) from the carbon DEPT spectra supported a pentacyclic tritepeniod sketal structure of a betuilene. The presence of two olefinic protons at 4.7 to 4.8 which were attached to a carbon at 106.1 ppm suggested a terminal CH_2_. The lack of a carboxylic carbon at 178-183 ppm indicated that it was not a triterpenic acid, but the carbon at 217.2 ppm suggested the presence of a ketone functional group and thereby confirms the structure of compound 1 as 3-Oxo-28-hydroxylbetuli-20(29)-ene with the aid of the 2D NMR (Figure 1). Furthermore, literature data as shown in Tables 3 and 4 correlates with our data and thereby confirms structure compound 1 as a 3-Oxo-28-hydroxylbetuli-20(29)-ene. 3-Oxo-28-hydroxylbetuli-20(29)-ene) was obtained by column chromatography as a white powder, it was visible under the UV light. NMR was used for characterization and structure elucidation. Calculated molecular formula C_30_H_48_O_2_ and Molecular weight of 440.

#### Compound 2: 3,28-dihydroxylbetuli-20(29)-ene

This was isolated as a colourless crystal powder. The ^1^H-NMR spectrum had the characteristic triterpenoid patterns with clusters of peaks around values 0.50 to 2.50 and similar spectra pattern as compound 1 suggested a triterpenoid compound. Analysis of the ^13^C-NMR spectra showed that it is a C30 compound which further strengthen the class of compound suggested. The ^1^H-NMR spectrum showed peaks at 3.58 (H-28); 4.70 and 4.80 (H-29) which is a hydroxyl group at position 28 and olefinic protons at position 29 which are similar to the proton chart of compound 1. There is also another peak at 4.20 which represent OH in the chemical shift chart. This suggested the present of an OH group at position 3 since there ketone carbon was absent.

**Figure 2:**
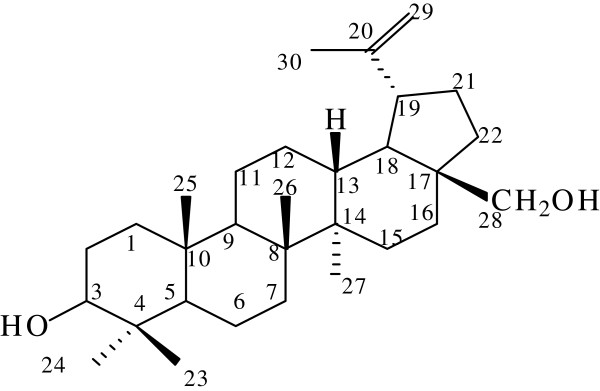
**Legend: elucidated structure of compound 2 (3,28-dihydroxylbetuli-20(29)-ene)** [24–26]**. δ**_**c**_ **(DMSO_d6)**: 14.93; 16.23; 18.18; 19.80; 21.43; 26.95; 31.82; 34.41; 35.71; 43.18; 47.08; 49.68; 54.55; 63.31; 106.18; 155.53; 217.23. **δ**_**H**_ **(DMSO_d6)**: 0.64; 0.76; 0.85; 0.86; 0.90; 0.94; 0.97;1.23; 1.33; 1.42; 1.43; 1.63; 1.97; 2.21; 2.21; 2.49; 2.95; 2.96; 2.97; 3.58; 4.26; 4.72; 4.75; 4.85.

The presence of six tertiary methyl (CH_3_), and one carbonyl carbon signals in the proton and carbon 13 spectra respectively; twelve methylene (CH_2_) signals, five methine (CH) and seven quaternary carbons (C) from the carbon DEPT spectra supported a pentacyclic tritepeniod sketal structure of a betuilene. The presence of two olenific protons at d 4.70 to 4.80 which were attached to a carbon at 106.1 ppm suggested a terminal CH_2_ (HSQC). The absence of a carboxylic carbon at 178-183 ppm indicated that it was not a triterpenic acid, and at 217.2 ppm suggested that the presence of a ketone functional group which was noticed in compound 1 was completely absence in compound 2 (Figure 2). Compound 2 was therefore elucidated to be 3,28-dihydroxylbetuli-20(29)-ene. Compound 2 was isolated as colourless powder during column chromatography. The elucidated structure was supported by the NMR spectra which showed double bond at position 29, dihydroxyl group in position 28 and 3.

## Discussion

*C. transvaalensis* and *V. infausta* showed good antibacterial activity against multidrug resistant*S. aureus* and *S. epidermidis*. Forty five percent and 58% of clinical strains of multidrug resistant *S. aureus* and *S. epidermidis* respectively were susceptible to *C. transvaalensis. V. infausta* inhibited 70% (*S. aureus*) and 36% (*S. epidermidis*). *C. transvaalensis* have been reported to have various biological activities [27–29]. Shai et al. [30] tested and confirmed antibacterial activity of *V. infausta* extracts against several bacterial species including Gram positive bacteria with MIC values of 0.2 mg/ml. It was also observed that *C. gratissimus* also had good activity against *S. epidermidis* because it was able to inhibit 58% of the clinical isolates. HIV is one of the most problematic Public Health concerns worldwide as it contributes as front liner cause of deaths among African population. The observed anti-HIV activity as well as minimal toxicity of these studied plants offer promise for their phytotherapeu1tic application in management of HIV/AIDS. In this study *C. transvaalensis* and *C. gratissimus* were able to inhibit the HIV infection growth.

The cellular cytotoxicity of extracts was screened in cells representative of the immune system especially those known to be the host cells for HIV infection. Therefore MT-4 cells were selected for both anti-HIV activity (EC_50_) using whole virus and cellular cytotoxicity screening (CC_50_). Out of four plant extracts evaluated for anti-HIV and cytotoxicity *Croton grattisimus* and *Cassine transvaalensis* exhibited SI values >5 which is an indication of antiviral activity with CC_50_ greater than 40 μg/ml which was greater than that of the berberine used as a positive control.

Compounds isolated from *C. transavaalensis* were found to be triterpenoids because they have 30 carbons and five rings. These compounds were identified by comparison of their spectroscopic data with those reported in literature [26] (Tables 3 and 4). Compound 1 has molecular formula of C_30_H_48_O_2_. The ^13^C-NMR spectra of compound 1 showed signal for CH_3,_ CH_2_,CH, HC = CH, C = O and quaternary C. ^1^H-NMR was used to detected the number of hydrogen atoms presence in the unknown compound which was white powder. The compounds isolated from *C. transvaalensis* were proven to be active against *S. aureus* and *S. epidermidis* because of the clear zone around them on the bio autographic assay. The R_f_ value of compounds isolated corresponded to that of the active compounds in the bio autographic assay. Compound 2 has a hydroxyl group at C-3 position in its structure which is a secondary hydroxyl group and a primary hydroxyl group at position C-28. The isolated triterpenes are also known to have anti-inflammatory and anti-HIV-1 activity [31]. The ^1^H-NMR spectrum showed peaks at 3.8 (H-28); 4.7 and 4.8 (H-29) which was a double bond which corresponded to the hydrogen position in the chemical shift chart. The six peaks at position 1.0 to 0.7 corresponded to the CH_3_ position in the chemical shift chart. The ^13^C-NMR detected the carbon atoms in compounds and showed the peak in position 217.2 was a ketone. The other peaks were found at position 15.5 (C_20_) and 106.18 (C_28_). The ^1^H-^13^C HSQC showed the corresponding atoms of hydrogen and carbon. It is seen that the two peaks at H-29 correspond to C-29 and also H-28 to C-28.

Tables 3 and 4 shows the ^1^H-NMR and ^13^C-NMR data of compound 1 and 2 which were assigned with the help of HSQC, COSY, HMBC and NOESY. Further comparism with literature. Literature has confirmed that these compounds have been isolated before from other plant sources hence the comparison in the above table.

## Conclusion

Data from this study supports the traditional use of studied plants in the treatment of skin ailments caused by staphylococci species. The results from this study also suggests that further screening of South African plants used in traditional medicine is warranted. *C. gratissimus, C. transvaalensis* and *V. infausta* can be considered as potential sources of antimicrobial agents against the test pathogens.
